# FuncTree2: an interactive radial tree for functional hierarchies and omics data visualization

**DOI:** 10.1093/bioinformatics/btz245

**Published:** 2019-04-20

**Authors:** Youssef Darzi, Yuta Yamate, Takuji Yamada

**Affiliations:** Department of Life Science and Technology, Tokyo Institute of Technology, Tokyo, Japan

## Abstract

**Summary:**

Functional annotations and their hierarchical classification are widely used in omics workflows to build novel insight upon existing biological knowledge. Currently, a plethora of tools is available to explore omics datasets at the level of functional annotations, but there is a lack of feature rich and user-friendly tools that help scientists take advantage of their hierarchical classification for additional and often invaluable insights. Here, we present FuncTree2, a user-friendly web application that turns hierarchical classifications into interactive and highly customizable radial trees, and enables researchers to visualize their data simultaneously on all its levels. FuncTree2 features mapping of data from multiple samples and several navigation features like zooming, panning, re-rooting and collapsing of nodes or levels.

**Availability and implementation:**

FuncTree2 is freely available at https://bioviz.tokyo/functree2/ as a web application and a REST API. Source code is available on GitHub https://github.com/yamada-lab/functree-ng.

**Supplementary information:**

Supplementary data are available at *Bioinformatics* online.

## 1 Introduction

Functional annotations are a common reference for summarizing the output of high-throughput experiments into a tangible format for downstream data analysis, making them the backbone of most omics computational workflows. More often than not, these annotations are complemented with a hierarchical classification that places them into a biological context, like metabolic pathways or broader functional definitions, and thus provides complementary insights to molecular level information. Adequate tools for visualizing functional annotation profiles simultaneously with their hierarchical classification not only simplify data interpretation but also help formulating more informed hypotheses or uncovering additional scientific insights. Yet this hierarchical classification is mostly limited to highlighting the trends of different levels separately onto pie and bar charts, thus losing the hierarchical information. As an alternative to these charts, a tree based representation, which displays the hierarchical structure together with quantitative charts, can overcome this limitation. Although the ubiquitous horizontal/vertical hierarchical tree layout is well suited for visual comparison, it scales poorly with large trees like functional hierarchies, as it does not use the drawing space effectively to represent a large number of nodes. On the other hand, radial trees offer a compact, yet comprehensive representation of a hierarchical classification, they scale to several thousands of nodes (Letunic and Bork, 2016), and are already familiar to life scientists (Asnicar *et al.*, 2015; Huson and Scornavacca, 2012; Letunic and Bork, 2016) which makes them an ideal medium for exploring functional classification. To date, the available tools implementing this concept are capable of visualizing only one sample at a time (Ondov *et al.*, 2011) or are limited to one predefined ontology or annotation source (Uchiyama *et al.*, 2015) which limits their potential. For a detailed feature comparison please refer to the Supplementary Material.

Here we present FuncTree2, a web application for visualizing functional hierarchies on radial trees and customizing them with one or multiple omics profiles. It features a highly interactive tree viewer that helps in exploring the trees, a REST API for automated access, and has a tab separated data matrix as the only input requirement.

## 2 Materials and methods

**Fig. 1.**
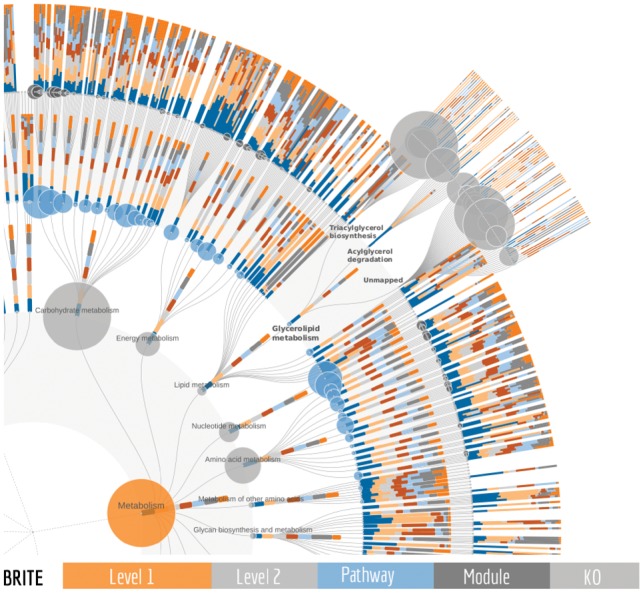
An example of a FuncTree2 output. Using a tab separated data matrix with seven samples and a sum column as input, FuncTree2 mapped them onto the KEGG BRITE tree which contains 5 levels and 13081 nodes. The sample abundances are visualized as stacked bar plots while the sum is visualized as circles, with the size reflecting the node abundance. Under the glycerolipid metabolism, three nodes are expanded to show KO level details for two modules and for KOs that are not part of a module

FuncTree2 turns a hierarchical classification file into an interactive tree using the D3 Radial Tidy Tree implementation (Bostock *et al.*, 2011). Each layer of nodes is mapped to a level of the hierarchy. The outermost layer is always the lowest level visualized, while the innermost layer is the highest level, and nodes represent biological entries (e.g. orthologous genes, pathways, biological processes) (Fig. 1). Global depth of the hierarchy can be adapted by controlling the ‘Max depth of tree’ in the customization panel, for collapsing or expanding layers. Nodes are also collapsible, using mouse clicks, to control the depth locally, and additionally can be re-rooted (right-click, then set as root) to focus on a specific level and its children. Zooming and panning as well as a search with autocomplete are available to help quickly locate a biological entry of interest.

FuncTree2 can visualize any functional classification after uploading it as a tab separated matrix or a JSON file compliant with the JSON Schema definition of a reference tree (for details please see the help https://bioviz.tokyo/functree2/docs/#json-reference-schema). However, by default, KEGG BRITE (Kanehisa *et al.*, 2014) and FOAM (Prestat *et al.*, 2014) functional hierarchies, which have KEGG Orthology (KO) annotations as the deepest level, are available as reference trees. Tab separated data matrices of omics profiles can be uploaded and reflected on these trees. For example, KO, eggNOG (Huerta-Cepas *et al.*, 2016), Gene Ontology terms (Ashburner *et al.*, 2000) or NCBI gene identifiers (NCBI Resource Coordinators, 2017) abundance from one or multiple samples, is visualized on the KO layer in the form of stacked bars or colored circles (Fig. 1). Values for parent nodes are derived by summarizing its children KO abundances into data series of sum and mean abundance and visualized in a similar fashion. Users can easily switch between the data series to visualize. Moreover, when metabolic modules (Kanehisa *et al.*, 2014) are available (e.g. KEGG BRITE) a module coverage can be added to the data series and used for visualization. In this case, on parent levels the mean of module coverage is used. Beyond its unique ability to visualize multiple samples, FuncTree2 is also the only tool that can display two types of data at the same time. For instance with an input matrix containing a column for the sum of rows, the sum could be visualized as circles which is particularly useful when sample abundances are visualized as bar charts of percentages instead of absolute values (100% stacking). Or in the case of an enrichment analysis, the *P*-values of one sample could be displayed as circles and its abundances as bars. In addition to the main radial tree, FuncTree2 summarizes layers into interactive bar plots, pie charts, heatmaps, and is complemented by an integrated iPath (Darzi *et al.*, 2018) viewer, an exclusive feature to FuncTree2 that provides seamless exploration of the data on pathway diagrams. All the customizations can be exported in high quality graphics like SVG, EPS and PDF.

Users with coding skills can take complete control of the customization by uploading a JSON file containing a customization for each node in the tree, after choosing the ‘Display’ action at the homepage or from the ‘Submit’ drop-down menu. Alternatively, the file can be submitted via its REST API which provides FuncTree2 as a visualization service for bioinformatic pipelines. For examples and detailed documentation please refer to the ‘Batch Access’ section of the online help.

## 3 Conclusion

FuncTree2 is a user-friendly web application that turns a hierarchical classification into an interactive radial tree explorer and offers users an advanced customization interface to visualize and explore their experimental data onto these trees, in a biologically relevant context. Unlike the current state of the art tools, it is capable of visualizing multiple samples, up to two types of data, and features additional built-in visualizations like iPath integration to bring complementary insights on the data and hopefully improves analysis outcome. Moreover, with simplified input requirements, built-in mapping, and web access, it lowers the technical barrier required for visualizing functional hierarchies. Finally, its REST API and ability to handle generic reference trees makes it an excellent tool for complementing bioinformatic pipelines with an advanced hierarchical data visualization service and to help exploring additional functional hierarchies.

## Funding

This work was supported by the Japan Science and Technology Agency PRESTO [JPMJPR1507]; and Japan Agency for Medical Research and Development (AMED) [17ek0109187h0002].

*Conflict of Interest*: none declared.

## Supplementary Material

btz245_Supplementary_Table_S1
